# ROR1 and ROR2 expression in pancreatic cancer

**DOI:** 10.1186/s12885-021-08952-9

**Published:** 2021-11-11

**Authors:** Dongli Liu, George Sharbeen, Phoebe Phillips, Australian Pancreatic Cancer Genome Initiative, Amber L. Johns, Anthony J. Gill, Lorraine A. Chantrill, Paul Timpson, Angela Chou, Marina Pajic, Tanya Dwarte, David Herrmann, Claire Vennin, Thomas R. Cox, Brooke Pereira, Shona Ritchie, Daniel A. Reed, Cecilia R. Chambers, Xanthe Metcalf, Max Nobis, Nicola Waddell, John V. Pearson, Ann-Marie Patch, Katia Nones, Felicity Newell, Pamela Mukhopadhyay, Venkateswar Addala, Stephen Kazakoff, Oliver Holmes, Conrad Leonard, Scott Wood, Sean M. Grimmond, Oliver Hofmann, Jaswinder S. Samra, Nick Pavlakis, Jennifer Arena, Hilda A. High, Ray Asghari, Neil D. Merrett, Amitabha Das, Peter H. Cosman, Kasim Ismail, Alina Stoita, David Williams, Allan Spigellman, Vincent W. Lam, Duncan McLeod, Judy Kirk, James G. Kench, Peter Grimison, Charbel Sandroussi, Annabel Goodwin, R. Scott Mead, Katherine Tucker, Lesley Andrews, Michael Texler, Cindy Forrest, Mo Ballal, David Fletcher, Maria Beilin, Kynan Feeney, Krishna Epari, Sanjay Mukhedkar, Nikolajs Zeps, Nan Q. Nguyen, Andrew R. Ruszkiewicz, Chris Worthley, John Chen, Mark E. Brooke-Smith, Virginia Papangelis, Andrew D. Clouston, Andrew P. Barbour, Thomas J. O’Rourke, Jonathan W. Fawcett, Kellee Slater, Michael Hatzifotis, Peter Hodgkinson, Mehrdad Nikfarjam, James R. Eshleman, Ralph H. Hruban, Christopher L. Wolfgang, Aldo Scarpa, Rita T. Lawlor, Vincenzo Corbo, Claudio Bassi, Andrew V. Biankin, Nigel B. Jamieson, David K. Chang, Stephan B. Dreyer, Caroline E. Ford

**Affiliations:** 1 Gynaecological Cancer Research Group, Lowy Cancer Research Centre, School of Women’s and Children’s Health, Faculty of Medicine & Health, University of New South Wales, Sydney, New South Wales 2052 Australia; 2 Pancreatic Cancer Translational Research Group, Lowy Cancer Research Centre, School of Medical Science, Faculty of Medicine & Health, University of New South Wales, Sydney, Australia; 3 Australian Pancreatic Cancer Genome Initiative (APGI), Sydney, NSW Australia

## Abstract

**Background:**

The Wnt receptors ROR1 and ROR2 are generating increased interest as cancer therapeutic targets but remain understudied in pancreatic ductal adenocarcinoma (PDAC). Compared to canonical Wnt/ β-catenin signalling, the role of noncanonical Wnt signalling in PDAC remains largely unknown. Only one study has investigated the prognostic significance of the noncanonical Wnt signalling receptor, ROR2 in PDAC. No studies have investigated the prognostic role of ROR1 in PDAC.

**Methods:**

Here, we performed analysis of ROR1 and ROR2 mRNA expression in three publicly available datasets ICGC-PACA-AU (*n* = 81), TCGA-PAAD (*n* = 150) and CPTAC-PDAC (*n* = 137). ROR1 and ROR2 protein expression from the CPTAC-PDAC discovery cohort were also analysed. Immunohistochemistry (IHC) using the validated anti ROR1 monoclonal antibody (4A5) was performed on the Australian Pancreatic Cancer Genome Initiative (APGI) cohort of PDAC samples (*n* = 152). Association between ROR1 cytoplasmic staining intensity and clinicopathological parameters including stage, grade and overall survival (OS) was investigated.

**Results:**

High ROR1 mRNA expression levels correlated with a favourable OS outcome in all of the ICGC-PACA-AU, TCGA-PAAD and CPTAC-PDAC cohorts. ROR1 protein expression was not associated with stage, grade or OS in the APGI cohort.

**Conclusion:**

ROR1 and ROR2 have potential as prognostic markers when measured at the mRNA level in PDAC. Our IHC cohort did not support ROR1 protein expression in predicting OS, and highlighted the discrepancy of prognostic biomarkers when measured by MS, IHC and RNAseq.

**Supplementary Information:**

The online version contains supplementary material available at 10.1186/s12885-021-08952-9.

## Background

Pancreatic cancer represents the 11th most common cancer worldwide with 458,918 new cases and 432,242 deaths recorded in 2018 [1]. It is also one of the most lethal malignancies, with 5-year survival rate less than 10% [2]. Risk factors associated with the disease include smoking [3], family history [4], germline mutations [5] and genetic mutations [6]. Despite the increased awareness around risk factors, the worldwide incidence and mortality of pancreatic cancer are both predicted to increase in coming years [7].

Pancreatic ductal adenocarcinoma (PDAC) is the most common type of pancreatic cancer that accounts for more than 80% of total cases. The majority of PDAC cases originate from microscopic precursor lesions called pancreatic intraepithelial neoplasia (PanINs) that are challenging to detect by current clinical imaging techniques [8]. As such, PDAC is typically diagnosed at an advanced stage (Stage III or IV) and presents an extremely poor prognosis compared to the less common pancreatic neuroendocrine tumour [9]. Current treatment options for patients are limited, especially for those with locally advanced or metastatic disease who are not eligible for surgery. In terms of chemotherapy, gemcitabine plus Abraxane has become the standard treatment based on moderate improvement outcomes compared to gemcitabine alone (median overall survival 8.5 months compared to 6.7 months, *p* < 0.001) from a phase III clinical trial [10]. Unfortunately, combination therapy with other agents shown to be effective in other cancers have not shown promising results in PDAC [11, 12]. In addition, immunotherapies, either alone or in combination with chemoradiotherapy or targeted therapy failed to show much progress in PDAC [13–15]. There is an urgent need of identifying biomarkers which could be targeted therapeutically for the malignancy.

Wnt signalling is one of the key pathways involved in cell differentiation, polarity, migration, adhesion and invasion. Encompassing both canonical (β-catenin dependent) and non-canonical (β-catenin independent) arms, the pathway has been implicated in a range of cancers. In PDAC, activation of the β-catenin dependent Wnt pathway has been indicated by cytoplasmic and nuclear localization of β-catenin [16], and suggested to play a role in progression of pancreatic cancer [17]. Mutations in *RNF43*, a gene associated with Wnt/ β-catenin pathway regulation have also been reported in PDAC [18, 19].

In contrast, the role of the β-catenin independent Wnt signalling arm remains largely unexplored in PDAC. The receptor tyrosine kinase-like orphan receptors ROR1 and ROR2 regulate β-catenin independent signalling and have been linked to carcinogenesis and metastasis in several malignancies [20–23]. A single study in PDAC (*n* = 162, ROR2 polyclonal antibody #LS-C99126, LifeSpan BioSciences Inc., USA) found high ROR2 protein expression was significantly associated with malignant characteristics and reduced overall survival [24]. An early study into ROR1 protein expression across a range of tumour types reported “moderate” or “high” expression in 83% of pancreatic cancer samples (*n* = 48) using the high-affinity 4A5 monoclonal antibody against ROR1 [25]. A separate study (using a separate C-terminus targeting monoclonal antibody) detected ROR1 protein at low levels in 15% of pancreatic adenocarcinoma samples (*n* = 38) [26]. Both studies also highlighted the presence of ROR1 expression in normal pancreatic islet cells.

The aim of this study was to clarify expression of ROR1 or ROR2 in PDAC through the interrogation of publicly available datasets and by performing immunohistochemistry (IHC) with a high-affinity monoclonal ROR1 antibody (4A5) on an independent and well-characterised cohort of PDAC [25, 27]. As there are numerous ROR1/2 targeting therapies in development [28, 29] and clinical trials [30–32] in progress, PDAC patients may benefit from this approach in the future.

## Methods

### Public datasets

The mRNA expression and accompanying clinicopathological data were acquired via three public platforms: International Cancer Genome Consortium (ICGC, https://icgc.org/),the Cancer Genome Atlas (TCGA, http://cancergenome.nih.gov/) and the Clinical Proteomic Tumor Analysis Consortium (CPTAC, https://cptac-data-portal.georgetown.edu/). Specifically, the normalised mRNA expression generated by next generation sequencing (NGS) was extracted for the ICGC-Pancreatic Cancer - Australia (ICGC-PACA-AU) cohort from the supplementary material of the corresponding publication [33]. Only cases with tumour purity above 40% indicated by the Qpurity score were included. For the TCGA-pancreatic adenocarcinoma (TCGA-PAAD) cohort, normalised gene expression of NGS was downloaded from the UCSC Xena platform [34] on 3rd May, 2020. The normalised gene expression data of the CPTAC cohort was obtained from the Genomic Data Commons (GDC) Data Portal (https://portal.gdc.cancer.gov/) on 2nd Sep, 2021.

In addition, the mass-spectrometry (MS) based proteomic data of the PDAC Discovery Study used in this publication were generated by the National Cancer Institute (NCI) CPTAC. We extracted relative abundance level of ROR1 and ROR2 protein (unshared log ratio) from the CPTAC data portal (https://pdc.cancer.gov/pdc/) on 2nd Sep, 2021.For all of the cohorts, only PDAC cases were included in the analysis. The characteristics of each cohort was shown in Table 1.

**Table 1:** Characteristics of the public datasets used in the study

		ICGC-PACA-AU [33]	TCGA-PAAD [18]	CPTAC-PDAC [35]
Total (n)^a^		81	150	137
Stage	I	6	13	4
	II	70	128	76
	III	1	4	36
	IV	4	4	7
Tumour grade	G1	1	20	9
	G2	45	85	97
	G3	31	42	30
	G4	2	1	1
Detection platform		Illumina HiSeq 2000	Illumina HiSeq 2000/2500	Illumina HiSeq (RNAseq) Thermo EASY-nLC 1200 UHPLC System (mass-spectrometry)

^a^Cases included in the analysis

### Clinical cohort

Tumour tissue microarrays (TMAs) and accompanying clinicopathological data of the Pancreatic Ductal Carcinoma Validation set (*n* = 334) were acquired from the Australian Pancreatic Cancer Genome Initiative (APGI). Immunohistochemistry (IHC) staining of ROR1 (1:50, #564464, BD Biosciences, USA) was optimised by the Kinghorn Cancer Centre Histopathology Laboratory at the Garvan Institute of Medical Research using the Leica Bond RX system (Leica Microsystems, USA). Negative control TMA slides (omission of primary antibody) were stained in parallel. The intensity of ROR1 staining was graded as 0 (absence), 1 (weak) and 2 (intense). Only the cases with three intact TMA cores (*n* = 157) were included, with the highest score set as the final score. We excluded another 5 from the final analysis for non-Pancreatic ductal adenocarcinoma (PDA) subtype, and ended up with the analysis cohort (*n* = 152). Demographic and clinicopathological parameters of the 152 patients were summarised in Table 2.

**Table 2:** Demographic and clinicopathological characteristics of the tumour samples in the APGI cohort

		Number of cases	Percentage (%)
Gender	Female	76	50.0
	Male	76	50.0
Tumour grade	1	8	5.3
	2	105	69.1
	3	39	25.7
FIGO stage (2009)	I	11	7.2
	II	128	84.2
	III	2	1.3
	IV	10	6.6
Recurrence status	Unknown	8	8.1
	Confirmed	91	91.9
Vital status	Alive	18	11.8
	Deceased of PC^a^	134	88.2

^a^PC- pancreatic cancer

### Statistical analysis

Both mRNA and protein expression levels were log2 transformed for all the tests. Statistical significance of expression level between tumour grades and stages was carried out using unpaired *t*-test in public datasets or Chi-square test in clinical cohort. Pearson’s correlation was performed to investigate relationship between mRNA and protein expression. Kaplan Meier curves were produced for univariate overall survival (OS) analyses. Optimal cutpoint for variables was determined by the maximally selected rank statistics from the ‘maxstat’ package in R. The log-rank test was used to evaluate the significance of the association. Cox multivariate regression including gender, stage and tumour grade was also applied on the OS. All the analyses were performed using R (v3.6.3). Figures were provided in R (v3.6.3) and GraphPad Prism (v7.02). Significance was set at *p* < 0.05.

## Results

### High ROR2 mRNA expression is associated with better overall survival in two independent PDAC cohorts

**Fig. 1:**
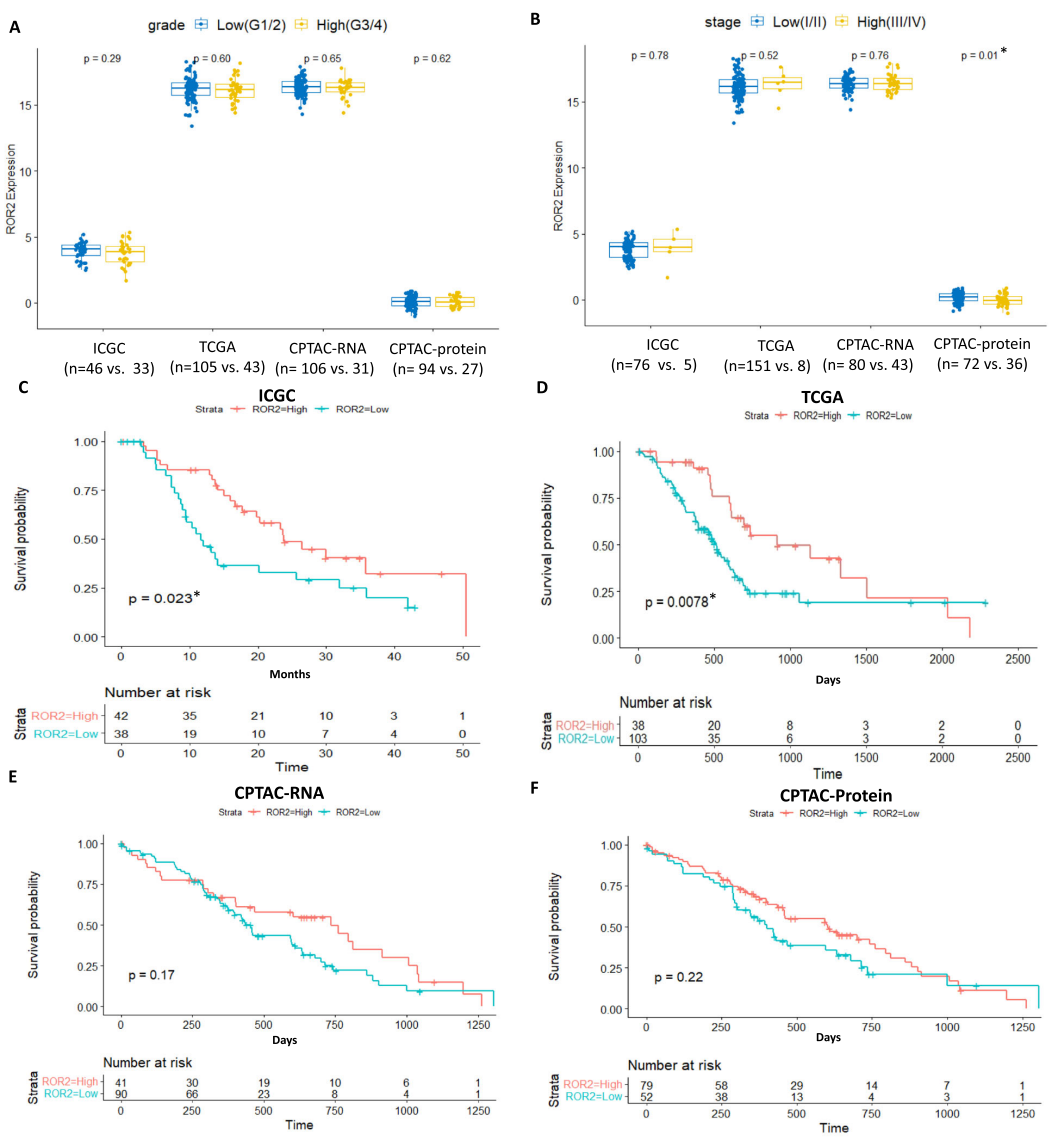
Association between ROR2 mRNA/protein expression level and clinicopathological parameters in the ICGC-PACA-AU,TCGA-PAAD and CPTAC-PDAC cohorts. A. No significant difference in ROR2 expression was observed between low and high tumour grade. B. ROR2 mRNA expression was not associated with stage, ROR2 protein expression was significantly different between low and high stage (*p* = 0.010). C. High ROR2 mRNA expression level was associated with better overall survival (*p* = 0.023) in the ICGC-PACA-AU cohort. D. High ROR2 mRNA expression level was associated with better overall survival (*p* = 0.008) in the TCGA-PAAD cohort. E. ROR2 mRNA was not associated with overall survival in the CPTAC-PDAC cohort. F. ROR2 protein was not associate with overall survival in the CPTAC-PDAC cohort.*Significance at *p* < 0.05 level

We correlated mRNA and protein expression of ROR2 with tumour grade, stage and overall survival (OS) in three independent pancreatic cancer cohorts (ICGC-PACA-AU, TCGA-PAAD and CPTAC-PDAC). No significant difference was observed for ROR2 mRNA expression between tumour stages or grades in either cohort (Fig. 1A,B). Lower ROR2 protein expression level was observed in high stage compared to low stage patients (*p* = 0.010, Fig. 1B). High ROR2 mRNA expression level was significantly associated with longer OS in both the ICGC and TCGA cohorts (*p* = 0.023 and 0.008 respectively, Fig. 1C,D), but not significant in the CPTAC-PDAC cohort (*p* = 0.170, Fig. 1E). ROR2 protein level was not significantly associated with overall survival based on the proteomic data of CPTAC-PDAC cohort (Fig. 1F).

### High ROR1 mRNA expression is associated with better overall survival in three independent PDAC cohorts

**Fig. 2:**
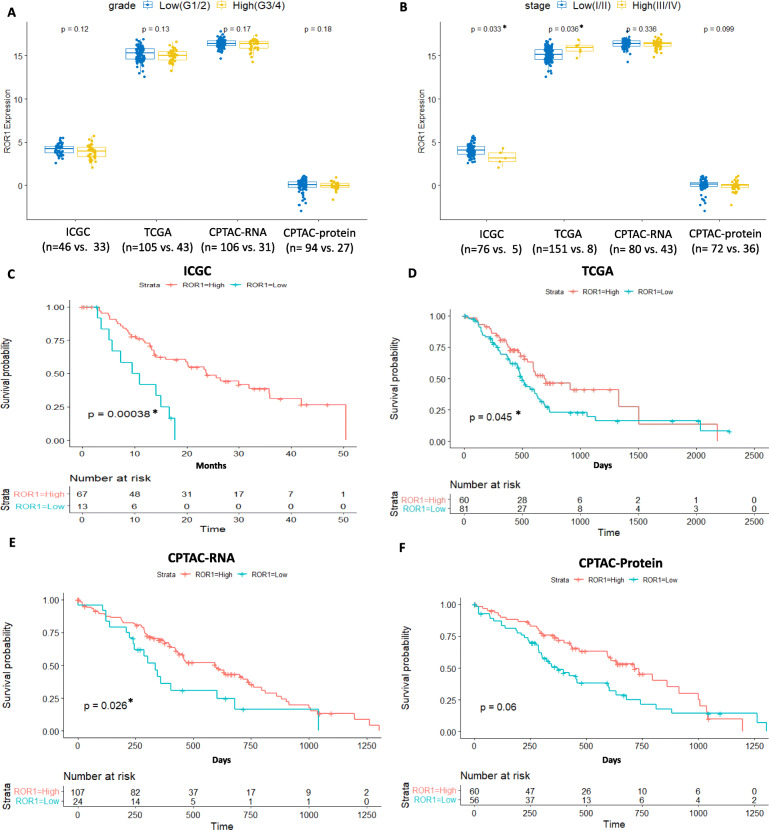
Association between ROR1 mRNA/protein expression level and clinicopathological parameters in the ICGC-PACA-AU, TCGA-PAAD and CPTAC-PDAC cohorts. A. No significant difference of ROR1 expression was observed between low and high tumour grade. B. ROR1 expression was associated with stage in ICGC-PACA-AU and TCGA-PAAD cohorts in opposite directions (*p* = 0.033 and 0.036 respectively). No significant correlation between ROR1 mRNA and stage was observed in CPTAC-PDAC cohort. C. High ROR1 mRNA expression level was associated with better overall survival (*p* < 0.001) in the ICGC-PACA-AU cohort. D. High ROR1 mRNA expression was associated with better overall survival (*p* = 0.045) in the TCGA-PAAD cohort. E. High ROR1 mRNA expression was associated with better overall survival (*p* = 0.026) in the CPTAC-PDAC cohort. F. ROR1 protein expression was not associated with overall survival in the CPTAC-PDAC cohort. *Significance at *p* < 0.05 level

We next correlated ROR1 mRNA expression with grade, stage, and OS in the cohorts. No association was seen between ROR1 mRNA or protein expression and grade in either cohort (Fig. 2A). ROR1 mRNA expression was lower in high stage (stage III or IV) versus low stage (stage I or II) in the ICGC cohort (Fig. 2B, *p* = 0.033) while the opposite trend was observed in the TCGA cohort (Fig. 2B, *p* = 0.036). However, both of the cohorts are limited in the number of high stage cases (*n* = 5 in ICGC and *n* = 8 in TCGA), which compromised the statistical power of the analyses. No significant correlation between ROR1 mRNA or protein level and tumour stage was observed in the CPTAC-PDAC cohort in which more late-stage samples were included (Fig. 2B). High ROR1 mRNA expression level was significantly associated with longer OS in all the three cohorts (*p* < 0.001, *p* = 0.045 and *p* = 0.026 respectively, Fig. 2C,D,E). However, the overall survival was not significantly associated with ROR1 protein expression level based on the CPTAC-PDAC cohort (Fig. 2F).

### ROR1 is not correlated with OS or clinicopathological parameters in PDAC at the protein level

**Fig. 3:**
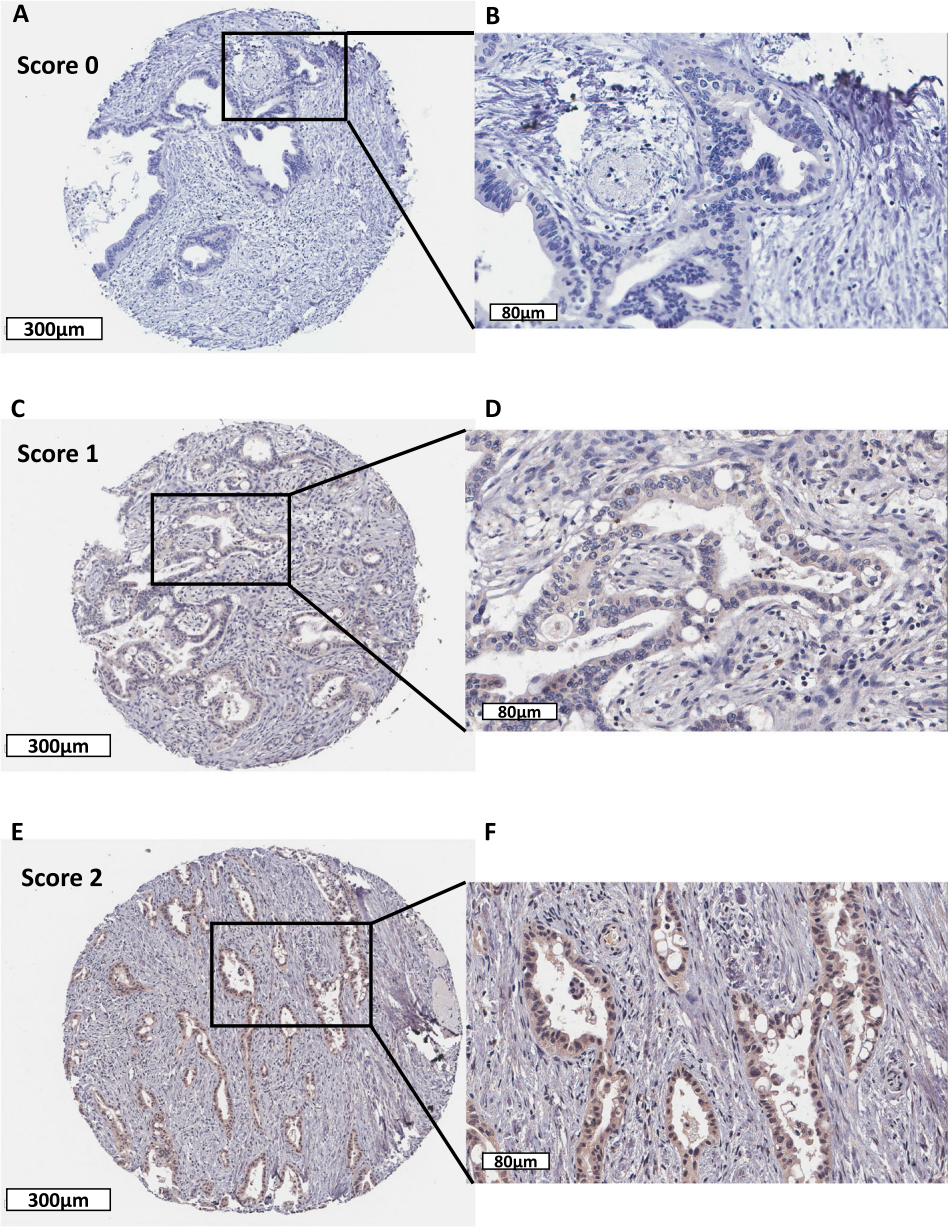
Representative immunohistochemistry images of ROR1 staining. Representative images of score 0 (absence; A,B), 1 (low; C,D) and 2 (high; E,F) for ROR1 expression measured by immunohistochemistry. Original magnification × 8 (scale bar = 300 μm) for A, C, E; magnification × 30 (scale bar = 80 μm) for B, D, F

**Fig. 4:**
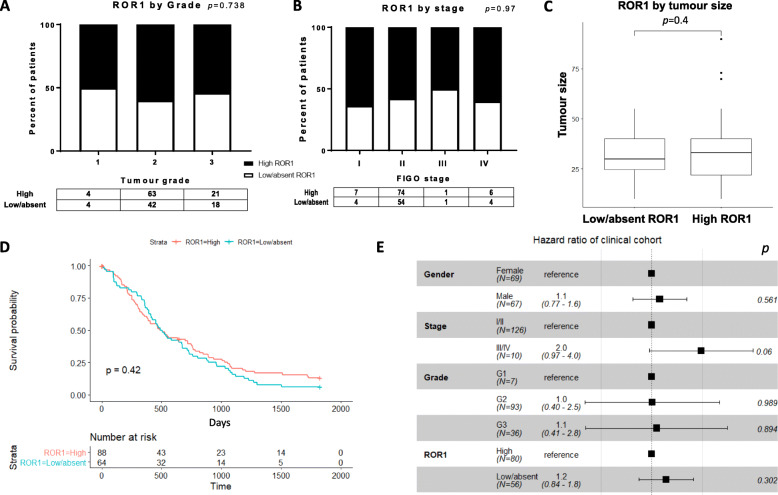
Correlation between ROR1 protein expression and tumour grade, stage, tumour size and overall survival in the APGI clinical cohort. A-C. Expression of ROR1 stratified by tumour grade, stage and tumour size. D. Kaplan-Meier analysis for ROR1 stratified by low/absent (score 0,1) and high (score 2). E. Multivariable overall survival analysis of ROR1 incorporating gender, tumour stage and grade

To investigate the role of ROR1 in PDAC at the translational level, we performed immunohistochemical staining of ROR1 in a well-defined cohort of 152 Australian PDAC tissue samples and correlated cytoplasmic expression with clinicopathological parameters and OS. Overall, the cohort showed a broad range of expression levels for ROR1 in PDAC tumour tissues (Fig. 3). ROR1 protein expression was not associated with tumour grade (Fig. 4A), stage (Fig. 4B) or tumour size (Fig. 4C), though it should be noted that the cohort is heavily skewed towards grade 2 and stage 2 tumours, with low numbers of samples from other grades and stages. No significant difference of OS was observed between high and low/absent ROR1 expression in terms of single or multivariable survival analysis (Fig. 4D,E).

## Discussion

The Wnt signalling receptors ROR1 and ROR2 are primarily expressed during embryogenesis and are downregulated in most adult tissues [36]. However, aberrant expression of ROR1 has been observed in several haematological malignancies and solid tumours including breast, ovarian and lung cancers [20, 22, 26, 37]. This tumour specific expression has made ROR1 a promising cancer drug target, leading to several ROR1 targeting therapies being developed. ROR1 targeting chimeric antigen receptors (CAR) T cell therapy has shown antitumour efficacy in vivo [38] and a humanised monoclonal antibody developed against ROR1 Fz and Ig-like domain, cirmtuzumab has demonstrated safety and efficacy in several Phase I/II clinical trials for patients with chronic lymphocytic leukemia (CLL), mantle cell lymphoma (MCL) (NCT03088878) and Her2-negative breast cancer (NCT02776917) [30, 31].

Two previous studies using independent ROR1 antibodies (4A5 and 6D4 monoclonal antibodies) have investigated ROR1 protein expression in small cohorts of pancreatic cancer tissues [25, 26]. While neither study investigated an association with overall survival, the detection of ROR1 protein expression prompted interest in pursuing ROR1 therapeutics in pancreatic cancer treatment. A small molecule KAN0439834 targeting the intracellular ROR1 TK domain induced significant apoptosis of ROR1 expressing PC cell lines [39], with additive effects observed in combination with erlotinib or ibrutinib. This raises the possibility of using ROR1 targeting therapies in future pancreatic cancer treatment. However, it should be noted that ROR1 protein expression was also detected in normal pancreatic tissue in previous studies [25, 26]. Off-target toxicity therefore needs to be considered when treating PDAC patients with ROR1-targeting therapies.

Ours is the first study to investigate an association between ROR1 protein expression and overall survival in pancreatic cancer patients. Previous studies of ROR1 expression via IHC reported cytoplasmic staining, even though ROR1 is a membrane receptor [25, 26]. We also detected ROR1 predominantly as cytoplasmic staining with the 4A5 antibody (same antibody used in [25]) in this cohort. It has been suggested that membranous staining is predominant if cells are fixed in paraformaldehyde while cytoplasmic expression more likely appears in formalin-fixed tissue [25]. ROR1 cytoplasmic staining was scored in tumour cells only, as no or low ROR1 was detected in surrounding stromal tissue. We observed no difference in overall survival in PDAC patients with low or high ROR1 expression in this cohort. We also saw no association with grade or stage in our cohort. However, it should be noted that this cohort (like most pancreatic cancer cohorts) is built from surgically resectable PDAC cases, which represents < 20% of PDAC patients. Tissue that makes its way into tumour biobanks and publicly available cohorts is often biased towards patients with a better overall prognosis. The majority of patients in our cohort were low grade (grade 1, 2 = 74%) and low stage (stage I, stage II = 92%).

At the transcriptional level, expression of ROR1 mRNA did associate with overall survival in three independent PDAC cohorts. All of the TCGA-PAAD, ICGC-PACA-AU and CPTAC-PDAC cohorts showed low ROR1 mRNA level was associated with worse OS compared to high ROR1 mRNA level. While two cohorts (TCGA and ICGC) showed divergent associations between ROR1 mRNA expression and stage, it should also be noted that both public cohorts consisted of only few high stage cases, which decreased the statistical power when analysing association between biomarker expression and stage in PDAC. As noted above, this is a consequence of cohort bias as only resectable tumours were biobanked. No significant correlation between ROR1 mRNA level and stage was observed in the CPTAC cohort which included a certain proportion of advanced staged cases (43 Stage III and IV out of total 137 cases). Apart from the limitation of resected PDAC samples, other differences in the cohorts could contribute to the discrepancy. One key difference could be the purity of tumour tissue biobanked and included in the separate mRNA expression analysis. PDAC tissue biopsies are typically composed of a significant proportion of stromal components, with low tumour cellularity commonly observed [18, 40]. Stromal and other cell contamination is likely to introduce noise that interferes with the gene expression analysis, particularly in the case of ROR1 where expression has been observed in healthy cells of the pancreas [25, 26]. One advantage of the ICGC cohort is the inclusion of tumour cellularity information for analysis. Tumour cellularity, the relative portion of tumour and normal cells of each sample in the cohort was estimated using the statistical model qpure [41]. In our analysis, we did not include any sample with epithelial content less than 40% from the ICGC cohort, which presented a more robust outcome than the TCGA cohort (median neoplastic cellularity 33% estimated with ABSOLUTE algorithm [18]). If we excluded the samples with tumour cellularity less than 40% in the TCGA cohort, we would lose statistical power for analysis dramatically due to a small sample size (*n* = 46). For the CPTAC cohort, all the cases included in this study had more than 40% cellularity. Other divergences among the cohorts were population origin, sample size, tumour grade distribution (Table 1), and mRNA expression cut-off.

Despite these limitations to individual cohort, it is unclear exactly why the prognostic significance of ROR1 expression at the mRNA level was not observed in ROR1 protein. A weak correlation between mRNA and protein expression (MS-based) was observed for ROR1 in the cases we included in CPTAC PDAC discovery cohort (R = 0.25, *p* = 0.005, Supplementary Fig.S1 A). Previous studies have reported a lack of direct relation between mRNA and protein expression [42, 43]. For ROR1, post-translational modifications such as glycosylation and mono-ubiquitination were observed extensively in CLL cells, which regulated the localization and stabilisation of ROR1 protein [44]. In addition, truncated ROR1 which lost the extracellular matrix and transmembrane regions was detected in central nervous system, leukemias and a variety of human cancers [45]. It is possible that individual isoforms of ROR1 could be undetected using the ROR1 monoclonal antibody we used in this cohort. Further studies are warranted to unravel the mechanisms behind the discrepancy between mRNA and protein level of ROR1 in PDAC.

Compared to ROR1, ROR2 has been less investigated as a cancer drug target, likely due to its complex dual role in carcinogenesis [46]. ROR2 has been reported to play either an oncogenic or tumour suppressor role based on different tumour context and appears more tightly linked to metastasis than proliferation [47–49]. Unlike ROR1, there are currently fewer ROR2 targeted drugs in clinical trials. A conditionally active biologic (CAB)-ROR2- antibody drug conjugate (ADC) named BA3021 was developed to selectively bind to ROR2 in the context of tumour microenvironment and has been reported to inhibit growth of ROR2 positive cell lines and xenografts of several cancers [50]. This drug is currently under Phase I/II trials for solid tumour, non-small cell lung cancer, triple negative breast cancer and soft tissue sarcoma (NCT03504488).

Our analysis of public datasets showed low ROR2 mRNA expression level correlated with worse OS in PDAC in the ICGC and TCGA cohorts (Fig. 1 C,D), which was contrary to the IHC staining results from a previous study [24]. For the ROR2 mRNA and protein level of the CPTAC cohort, no significant association was observed with OS (Fig. 1 E,F). That specific study reported that high ROR2 protein expression, indicated by both stromal and cytoplasmic staining, was correlated with worse OS and stage in a cohort of 162 PDAC cases. They used a polyclonal antibody against ROR2, and it should be noted that the validity of a number of ROR2 antibodies has been shown to be questionable [51]. We strongly recommend an independent assessment of ROR2 protein expression in a pancreatic cancer cohort to clarify this question.

In conclusion, ROR1 appears to be promising biomarkers in predicting survival outcome of PDAC patients at the mRNA expression level. However its use as protein biomarkers remains unclear. Our IHC analysis in a large well-defined PDAC cohort as well as the proteomic data of the CPTAC PDAC discovery cohort does not support the use of ROR1 as a prognostic biomarker at protein level in PDAC and highlights the discrepancy of prognostic biomarkers when measured by MS, IHC and RNAseq. The mechanism underlying the insufficient correlation between mRNA and protein level of ROR1 and ROR2 in PDAC remains unclear and warrants future investigation.

## Supplementary Information

**Additional file 1 **: **Supplementary Fig.S1**. Correlation between mRNA and protein expression of ROR1 and ROR2 in CPTAC PDAC discovery cohort. A. ROR1 mRNA and protein expression was positively correlated (Pearson’s *R*=0.25, *p*=0.005). B. ROR2 mRNA and protein expression was positively correlated (Pearson’s *R*=0.48, *p*<0.001).

## Data Availability

The datasets analysed during the current study are available in the International Cancer Genome Consortium (ICGC, https://icgc.org/), the Cancer Genome Atlas (TCGA, http://cancergenome.nih.gov/) and the National Cancer Institute’s Clinical Proteomic Tumor Analysis Consortium (CPTAC, https://pdc.cancer.gov/pdc/).
