# Supplementary material for: ROR1 and ROR2 expression in pancreatic cancer
Source: BMC Cancer. 2021 Nov 11;21:1199. doi: 10.1186/s12885-021-08952-9 (PMC8582180; doi:10.1186/s12885-021-08952-9)
Supplement: Supplementary file 1 — Additional file 1 : Supplementary Fig.S1. Correlation between mRNA and protein expression of ROR1 and ROR2 in CPTAC PDAC discovery cohort. A. ROR1 mRNA and protein expression was positively correlated (Pearson’s R=0.25, p=0.005). B. ROR2 mRNA and protein expression was positively correlated (Pearson’s R=0.48, p<0.001). [file 12885_2021_8952_MOESM1_ESM.docx]

**
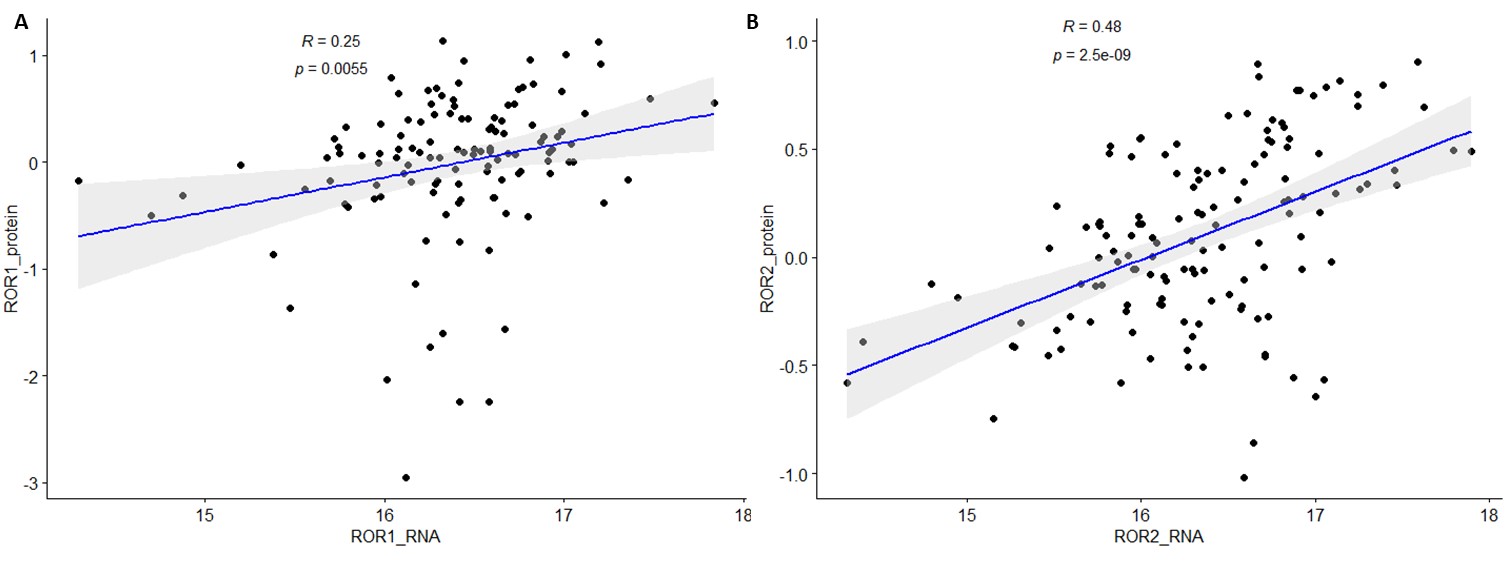
**

***Supplementary Fig.S1. Correlation between mRNA and protein expression of ROR1 and ROR2 in CPTAC PDAC discovery cohort.*** *A. ROR1 mRNA and protein expression was positively correlated (Pearson’s R=0.25, p=0.005). B. ROR2 mRNA and protein expression was positively correlated (Pearson’s R=0.48, p<0.001)*
